# Qualitative Assessment of Challenges in Tuberculosis Control in West Gojjam Zone, Northwest Ethiopia: Health Workers' and Tuberculosis Control Program Coordinators' Perspectives

**DOI:** 10.1155/2016/2036234

**Published:** 2016-03-15

**Authors:** Senedu B. Gebreegziabher, Solomon A. Yimer, Gunnar A. Bjune

**Affiliations:** ^1^Amhara Regional State Health Bureau, P.O. Box 495, Bahir Dar, Ethiopia; ^2^Institute of Health and Society, University of Oslo, P.O. Box 1130, Blindern, 0318 Oslo, Norway; ^3^Oslo University Hospital, 0424 Oslo, Norway; ^4^Norwegian Institute of Public Health, P.O. Box 4404, Nydalen, 0403 Oslo, Norway

## Abstract

*Background.* Weak health systems pose many barriers to effective tuberculosis (TB) control. This study aimed at exploring health worker's and TB control program coordinator's perspectives on health systems challenges facing TB control in West Gojjam Zone, Amhara Region, Ethiopia.* Methods.* This was a qualitative descriptive study. Eight in-depth interviews with TB control program coordinators and two focus group discussions among 16 health workers were conducted. Purposive sampling was used to recruit study participants. Thematic analysis was used to identify and analyse main themes.* Results.* We found that intermittent interruptions of laboratory reagents and anti-TB drugs supplies, absence of trained and motivated health workers, poor TB data documentation, lack of adherence to TB treatment guideline, and lack of access to TB diagnostic tools at peripheral health institutions were challenges facing the TB control program performance in the study zone.* Conclusions.* Ensuring uninterrupted supply of anti-TB drugs and laboratory reagents to all health institutions is essential. Continuous refresher training of health workers on standard TB care and data handling and developing and implementing a sound retention strategy to attract and motivate health professionals to work in rural areas are necessary interventions to improve the TB control program performance in the study zone.

## 1. Introduction

Tuberculosis (TB) remains a major threat to human beings, with the majority of cases occurring in the developing world. The burden of TB has shown significant decline over the past two decades. The implementation of the Stop TB strategy has played a key role in the global reduction of the TB burden. As reported by the World Health Organization (WHO), an estimated 43 million lives have been saved between 2000 and 2014. The Millennium Development Goal target of halting and reversing TB incidence has been achieved on a global scale. Mortality from TB has fallen by 47% since 1990 [1]. Despite these gains, however, TB still remains a major global health threat. According to a recent WHO report, there were 9.6 million new TB cases and 1.5 million deaths from TB worldwide [1]. The 22 high-TB burden countries collectively accounted for 80% of all estimated incident cases.

Ethiopia is among the 22 high-TB burden countries in the world. The directly observed treatment short course strategy (DOTS) has been implemented in the country since 1994 to control the TB epidemic. The prevalence, incidence, and mortality from TB in the country are currently estimated at 200/100,000 people, 207/100,000 people, and 33/100,000 people, respectively [1]. These indicators show that the TB burden in Ethiopia is still enormous and suggest that the National TB Control Program should be functioning very well to achieve significant reduction in morbidity and mortality resulting from TB.

Recently, a study was conducted in West Gojjam Zone of Amhara Region, Ethiopia, to assess trends in case notifications and treatment outcomes [2]. The study revealed a number of interesting results that require further assessment. Among others, poor TB data documentation, poor sputum smear examination follow-ups at the 2nd, 5th, and 7th months of treatment, and considerable proportion of deaths were observed during the study period. These findings may indicate that there were challenges affecting the success of the TB control program performance in the study zone. Weak health systems pose many barriers to effective TB control [3]. Understanding the challenges affecting the TB control program is crucial to improve performance and ultimately reduce the TB burden.

Various studies in Ethiopia and other countries investigated TB control program challenges from patients' perspectives [4–10]. However, the number of studies that explored the challenges from health worker's and TB control program manager's perspectives is limited [11–13]. Challenges facing TB control program may vary within different context. Lack of actual understanding of the challenges in context would limit subsequent improvement. Therefore, in-depth understanding of the challenges affecting the TB control activities is crucial to identify potential areas for improvement. This study was thus conducted to explore health worker's and TB control program coordinator's perspectives on health systems challenges facing the TB control program performance in West Gojjam Zone, Amhara Region, Ethiopia.

## 2. Materials and Methods

### 2.1. Setting

**Figure 1:**
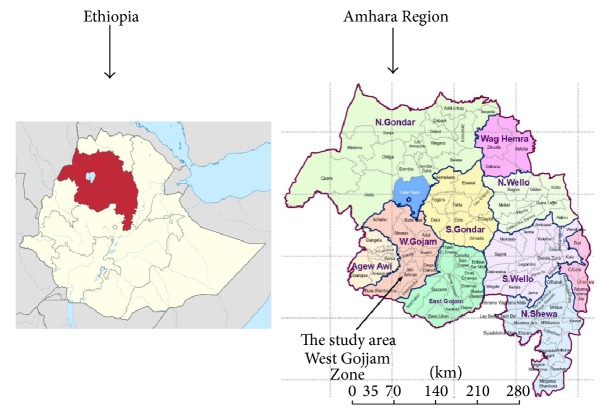
Map of Ethiopia, Amhara Region, and the study zone.

The study area, West Gojjam Zone, is one of the ten zones in the Amhara Region of Ethiopia (Figure 1). The total population is estimated at 2,382,497 million and more than 90% of the people reside in rural areas [14]. Agriculture is the main source of livelihood for the community. The zone covers an area of 13,669 Km^2^ and is administratively divided into 13 rural districts and five town administrations.

Health service in West Gojjam Zone is provided by one government hospital, 90 health centers, 76 private health facilities (hospitals and higher clinics), and 363 health posts. TB control program is decentralized in all districts and town administrations of the zone. One government hospital, 73 government health centers, and six private health institutions have TB diagnostic and treatment facilities. In addition, 276 health posts in the study zone are serving as treatment centers for implementing DOTS. A health post is the lowest level health facility and is staffed by two female health extension workers (HEWs). HEWs play an important role in identifying and referring TB suspects to the next level of health care, that is, health centers for diagnosis and initiation of treatment. Health posts are not equipped with TB diagnostic tools.

Sputum smear microscopy is the only TB diagnostic tool used in peripheral health centers. Thus, all sputum smear-negative pulmonary TB (PTB-) and extrapulmonary TB (EPTB) cases must be referred to hospitals and private clinics for chest radiography and other necessary investigations for TB.

### 2.2. Study Design, Participants, and Data Collection

This was a descriptive qualitative study conducted from January 2015 to March 2015. Qualitative data were collected using individual interviews and focus group discussions (FGDs). The in-depth interviews were conducted with eight TB control program coordinators who were recruited based on their experience in TB control program management at district, zonal, and regional levels. Two participants were recruited from Town Administration Health Offices, four were selected from District Health Offices, one was represented from West Gojjam Zone Health Department, and another one was selected from Amhara Regional State Health Bureau.

In-depth interview guide with open ended questions was employed to generate the relevant data. Key issues discussed were the regularity of anti-TB drug and laboratory supplies, availability of trained health workers at health facilities, and data quality in TB control program. In addition, respondents were requested to describe their perceptions regarding other challenges facing the TB control program performance in their context. The interviews were continued until saturation was reached. Saturation was reached during the 7th interview and there was no new information that was coming out other than repetition. The 8th interview was conducted to only verify whether there could be any new insights that might emerge, but this was not the case. All interviews were undertaken face-to-face. To ensure privacy and comfort, the interviews were conducted in a place convenient to the participants. The in-depth interviews were conducted by the principal investigator.

After the in-depth interviews were completed, preliminary analysis was conducted and major themes emerging from the data were identified. Subsequently, two FGDs were conducted with a total of 16 health workers. Eight participants took part in each discussion. Participants for the FGDs were selected with the assistance of health programs manager at the Zonal Health Department and TB control program coordinators at zonal, district, and health facility levels. Participants were purposively selected based on the responsibilities they have in relation to TB control activities at the respective health facilities where they were selected. The selected participants included were health workers involved in TB diagnosis and treatment. They were focal persons assigned for TB related activities at TB clinics and outpatient, laboratory, and pharmacy departments. In order to get a broad insight into the subject under study, professional diversity was considered in the composition of selected participants. Accordingly, four laboratory technologists, three pharmacy professionals, four health officers, and five nurses were recruited for the study. Participants were selected from health institutions (hospital and health centers) located in town administrations and rural districts of the study zone. A FGD guide was used and similar topics were explored in all the sessions. Issues that arose in the in-depth interviews were brought up during the FGDs for further discussion. We used this triangulation of data sources in order to increase the validity of findings in our study. Additional information was not coming out in the second FGD other than repetition of ideas.

Throughout the interviews and FGDs, follow-up questions using probes were asked in order to acquire a deeper understanding when an explanation was unclear. The interviews and FGDs were conducted in Amharic (the local language) for all participants. Each in-depth interview and FGD on average lasted 60 and 120 minutes, respectively, and was audio-taped with the consent of participants. All participants of the FGD hold a B.S. degree in their professions. The principal investigator and a trained assistant facilitated the FGDs. All participants actively participated and freely discussed the challenges facing the TB control program in their context.

### 2.3. Analysis

The audio-taped in-depth interviews and FGDs were transcribed verbatim and translated into English. The principal investigator thoroughly read the transcripts several times until she became familiar with the data. Thematic analysis was used to identify and analyse important themes [15]. The analysis focused on developing coding categories where narrative information was organized according to emerging themes. NVivo version 10 computer software program was used to code the transcripts. Coding of the data was done without fitting it into a preexisting coding frame. Equal attention was given to all data sets during coding. All coded data relevant to each theme was collated into potential themes. The themes that were identified through coding were further refined and developed. Quotes from respondents were included in the text in order to illustrate the findings.

### 2.4. Ethics

The Regional Committee for Medical Research Ethics (REK Øst) in Eastern Norway and the Ethiopian Ministry of Science and Technology in Ethiopia approved this study. Permission was obtained from respective local administrations. Informed-verbal consent was obtained from all participants before data collection. Confidentiality was ensured by not disclosing the identity of participants.

## 3. Results

Intermittent supply of anti-TB drugs, intermittent supply of laboratory reagents, trained health workers shortage, poor TB data documentation, and inadequate diagnostic tools were the most important themes identified from the analysis. Other themes identified were poor commitment of health workers and lack of adherence to TB treatment guideline. The themes are described below in detail with illustrative quotes from the data.

### 3.1. Intermittent Supplies of Anti-TB Drugs

As participants described, the Pharmaceuticals Fund and Supply Agency (PFSA) has been supplying anti-TB drugs to health facilities in the study zone. Health workers working in the TB clinics, the pharmacy professionals, and some of the TB control program coordinators mentioned that anti-TB drugs were intermittently supplied to health facilities. Participants also considered this issue as a common problem in their area.

Illustrations of this idea were mentioned as follows.

*There is TB drugs shortage, specifically pediatric fixed drug dose combinations are not regularly supplied. The supply is also not balanced. If ethambutol is provided, rifampicin, isoniazid, pyrizinamide (RHZ) may not be there. If RHZ is provided, ethambutol may not be supplied. There was a time that we treated children using adult form tablets of these drugs. (Participant #10)*

Health workers working in TB clinics expressed their concern about shortage of drugs for IPT. They described that isoniazid is recommended to children under five years of age who have contact history with smear-positive pulmonary TB patients. However, they reported that they had been experiencing shortage of IPT.

*Isoniazid should be given for all families of smear-positive pulmonary TB patients especially for children who are under the age of five years. However, there is no adequate supply of IPT. Previously, we had borrowed from Wegedad health center since we did not have the drug in our health center. (Participant #12)*

Participants were asked to clarify why supplies of anti-TB drugs were interrupted. They mentioned that PFSA did not timely deliver supplies to health facilities. In addition, TB control program coordinators stated that health facilities did not timely send drug consumption reports to PFSA based on the integrated pharmaceutical logistics system (IPLS) guideline. They also did not timely request the required amount of supplies for health facilities.

### 3.2. Intermittent Supplies of Laboratory Reagents

In addition to anti-TB drugs, intermittent supplies of reagents for performing Ziehl-Neelsen staining for acid-fast bacilli (AFB) were reported by majority of the TB control program coordinators and laboratory technicians. This was mentioned as a challenge for failing to regularly perform follow-up AFB microscopy test for sputum smear-positive cases. Participants were asked to clarify why laboratory reagent supplies for AFB test were interrupted. They mentioned the same reasons given for intermittent anti-TB drug supply as described above.

Illustrations of this idea were mentioned as follows.

*I was transferred to this health center before six months. There is great reagent shortage. We reported this to the district TB focal person. We also told the store man at the health center. In addition, health workers working at the outpatient department are complaining about this. There is a great reagent shortage for TB test. (Participant #14)*

*To get supplies at the right season, health centers must provide consumption reports for the previous supplies. We have provided training to health workers about IPLS and we are regularly discussing this issue when we go there for mentoring support. But sometimes, because of delay in sending consumption reports, laboratory reagent supplies are interrupted. (Participant #5)*

Some of the TB program coordinators reported that the follow-up sputum smear examination was not done regularly. Laboratory professionals also acknowledged the gaps in doing follow-up sputum smear examination for smear-positive pulmonary TB cases. Workload and lack of reagents were the suggested reasons for not regularly performing patient follow-up sputum smear examination.

*After completing the intensive phase therapy and before the continuation phase treatment begins, follow-up sputum smear examination should be done, but sometimes, either unknowingly or when they were busy or due to other reasons, health workers were not performing follow-up sputum smear examination for patients. (Participant #2)*

### 3.3. Trained Health Workers Shortage

As mentioned by majority of the participants, there were no adequate numbers of trained health workers for TB control program in most health facilities of the study zone. Some of the TB program coordinators reported that, due to lack of trained health workers, untrained health workers were assigned in TB clinics.

*In the past, untrained health workers were assigned in TB clinics. Untrained health workers do not properly know how to give the treatment, how to follow patients and when follow-up AFB examination should be done for TB patients. (Participant #11)*

Majority of the participants mentioned that a partner organization called Management Sciences for Health (MSH) that is implementing a project entitled “Help Ethiopia Address the Low Tuberculosis Performance (HEAL-TB)” in the study zone has been providing TB training to health workers. However, due to workload and high turnover of staffs in the health facilities, some of the health workers said that the number of available trained health workers was not adequate and suggested that standard TB training must be given for all health care providers to address this challenge.

*I don't think the available trained health workers are adequate for our health center. We are providing a number of health services, even dispensing TB drugs takes half a day. (Participant #9)*

*In places where two or more trained health workers are available, better services are provided, while in health centers where only one trained health worker is present, the TB service may be interrupted whenever the health worker is absent for various reasons. (Participant #2)*

Most participants expressed their concerns about the high turnover of trained staff in all health programs including TB control. Looking for work in health facilities located in urban areas and opting for further education and better payment were suggested reasons by most participants when they were asked to explain why high turnover of staff occurred.

*The turnover of staff is high and frequent. Whenever health workers get better opportunities, they may not stay in their current job. This is their right! We cannot force them. Unless TB training is given regularly, the problem will continue. (Participant #1)*

Lack of laboratory technicians at some rural health facilities of the study zone was a concern mentioned by some of the TB control program coordinators. They said that they had posted vacancy to hire laboratory personnel for these health facilities; however, nobody showed up to compete for the post. Due to lack of laboratory personnel, laboratory services have not been started in some rural health facilities of the study zone.

*For the time being, due to lack of laboratory personnel, we are not providing TB diagnostic service at four health centers. (Participant #2)*

Lack of interest in working in rural areas was the suggested reason for not finding applicants for the vacant post.

*In our zone, there are remote areas such as Sekela, Damot, Quarit, Wonberma, North Achefer and Gonji. Health workers may not want to go and work there, but we are trying our very best to encourage them to go there and work. (Participant #1)*

### 3.4. Poor TB Data Documentation

The TB control program coordinators reported poor recording and reporting of TB related activities at health facility level. They said that health workers do not give enough attention for data management and do not consider recording activities as part of their responsibilities. Lack of capacity on data management and lack of attention were the suggested reasons for poor TB data recording and reporting. TB control program coordinators further stated that they regularly discussed the problem with health care providers during supportive supervision and review meetings; however, they noted that the problem of poor data quality is still persisting.

The following was the illustration.

*Generally, there is a gap in monitoring and evaluation activities at all levels. Specifically to TB, there is a problem on data quality in recording and reporting of treatment outcomes. We have tried to solve this through supportive supervision, but even now, we are not sure to say that the data quality has been improved. (Participant #5)*

### 3.5. Lack of Diagnostic Tools

Few TB control program coordinators reported unavailability of diagnostic tools for diagnosing PTB- and EPTB cases at peripheral health institutions. They mentioned that smear microscopy has been used to diagnose smear-positive pulmonary TB cases in health centers. They further explained that suspected PTB- and EPTB cases are referred to the nearby hospitals and private clinics where better diagnostic tools are available. The TB program coordinators perceived lack of access to diagnostic tools at peripheral health facilities as one of the contributing factors to low TB case detection.

*We are examining TB patients mostly using a microscope, however, microscope's sensitivity is very low i.e. 40–60%. Due to this, we can only diagnose 40–60% of the TB cases out of 100 patients screened for active TB. The rest of the patients can be examined using culture, chest X-ray and other methods. But in our region, only government hospitals and limited number of private health institutions have chest X-ray facilities. (Participant #5)*

### 3.6. Health Workers Commitment

Health workers' commitment was a concern mentioned by participants. There were divergent views among them about health workers' dedication in performing TB control program activities. Some of the TB program coordinators perceived that some health workers lack commitment and are not devoted to their job. They complain of the workload they have. In contrast, majority of the participants described that, despite the workload, health workers are doing their jobs with good commitment. The study participants felt that health workers are at risk of TB infection unless they prevent and control the disease. Moreover, they noted that their families may be at risk of infection if they are infected. Some participants also mentioned that many health professionals have become very concerned about their job after the emergence of MDR-TB.

*If health workers don't work properly on TB prevention and control activities, they will be affected by the disease. The main transmission of TB infection is through air. Patients are coming to health facilities to seek care, unless we treat them, we can't stop disease transmission. (Participant #5)*

### 3.7. Lack of Adherence to TB Treatment Guideline

Majority of the participants generally acknowledged the necessity of strict DOT in TB management. However, they mentioned that TB treatment provision was not done according to the national TB treatment guideline. For example, when patients complained of showing up on daily bases at health facilities to take their anti-TB medication, some health workers were just providing them with anti-TB medications to take them at their home without being supervised by either a health worker or treatment supporter. Majority of the TB control program coordinators mentioned health worker's negligence as a reason for not adhering to the treatment guideline.

*There are principles to follow when providing ant-TB drugs during the two phases i.e. intensive and continuation phase treatment. However, health workers do not practice the treatment guideline according to DOTS. There is a problem of adhering to DOTS during the intensive phase, in particular. (Participant #17)*

*There is patient follow-up problem. In DOTS, anti-TB drugs must be taken daily throughout the intensive phase treatment under health worker's direct supervision, but when patients sometimes complain of different reasons, health workers dispense TB drugs to patients so that they can take it at home on their own. In addition, patients complete their treatment without having the AFB follow-up test. (Participant #11)*

## 4. Discussion

This descriptive qualitative study explored health workers' and TB control program coordinators' views on challenges facing the TB control program performance in the study zone. Most of the challenges the participants described were health system based. We suggested possible measures that may help to overcome these challenges in the study zone.

### 4.1. Intermittent Supplies of Reagents and Anti-TB Drugs

As reported by TB control program coordinators and laboratory technicians, there were interruptions in the supply of AFB reagents. Shortage and interruptions of AFB reagents supplies were reported from previous studies [12, 13, 16]. Sputum smear microscopy is widely used for TB diagnosis and monitoring of treatment progress in the study area [17]. Treatment monitoring is an essential component of TB treatment. Patient response to the treatment regimens is evaluated in the 2nd, 5th, and 7th month of treatment and corrective measures are taken based on the results. Continuous supply of reagents to all health facilities is mandatory to perform this task. Health facilities that lack reagents cannot monitor patient treatment response according to the treatment monitoring guideline, and this may increase the risk of unfavorable treatment outcome, that is, death, treatment failure, and also dissemination of drug resistant TB. In addition, intermittent reagents supplies may affect the routinely performed AFB smear microscopy test to diagnose patients in health facilities. This may discourage patients who seek health care and subsequently contributes to delay in diagnosis and treatment and increased transmission of TB.

Intermittent supplies of anti-TB drugs were also reported as a challenge. Patients' adherence to treatment is seriously affected in such conditions [18, 19]. It could also delay treatment initiation after diagnosis has been made and subsequently results in severe illness and increased transmission of TB. The National TB Control Program recommends isoniazid preventive therapy (IPT) for children [20]. Due to shortage of isoniazid, however, health care providers could not implement IPT. IPT has high efficacy in reducing mortality [21] and risk of developing TB among exposed children [22].

As part of a major intervention to improve the supply chain management in Ethiopia, PFSA, in partnership with its collaborators, has been implementing integrated pharmaceuticals logistics system (IPLS) since 2009 [23]. Failure to timely report consumption status and requesting new supplies based on IPLS guideline were reasons for irregular supplies of anti-TB drugs and AFB laboratory reagents in the study zone. Routine monitoring reports show that IPLS is improving information recording and reporting and storage and distribution systems as well as the availability of essential commodities at service delivery points [24]. Inappropriate implementation of IPLS may contribute to irregular supply of anti-TB drugs and reagents. Our findings suggest that strengthening IPLS implementation is needed to ensure uninterrupted anti-TB drugs and laboratory reagents supplies to health facilities. Enhancing a dialogue between health workers who are responsible for reporting and requesting anti-TB drugs and reagents and the PFSA staffs who are accountable for ensuring continuous supply of the requested supplies may help to solve the problem.

### 4.2. Trained Health Workers Shortage

As mentioned by the TB control program coordinators, there were conditions where TB patients had been treated by health workers who had not received standard TB training. Previous studies from Ethiopia and other countries highlighted this experience [13, 25–27]. Due in part to the high turnover and lack of adequate number of trained health workers, health facilities were providing TB care by untrained staffs. Without adequate TB training, health care workers are not able to provide proper counseling to TB patients and this will have a negative effect on patient adherence to treatment [26]. Managing TB patients by inadequately trained health workers could generally compromise the quality of TB care and may contribute to poor treatment outcome. This finding indicates that measures should be taken for ensuring the availability of adequate amount of trained health care workforce for TB at all times.

In addition to this, this study highlighted lack of laboratory personnel in some rural health facilities of the study zone. As mentioned by the TB control program coordinators, the District Health Offices had allocated the required budget to hire laboratory technicians. However, the remoteness of the health facilities seemed to have been the reason for laboratory technicians not applying for the vacant job. This may impede accessing TB diagnostic services to the rural community. Adequate number of skilled and motivated health workers at the right place and time is critical to deliver effective health services [28]. We suggest that the study Zone Health Department and the Amhara Regional State Health Bureau have to develop and implement a sound retention strategy that may attract and motivate health workers to work in rural and remote health facilities.

### 4.3. Poor Recording of TB Activities

TB control program coordinators acknowledged poor recording of TB activities as a challenge. Evidence from a recently conducted records review in the study zone supports this opinion [2]. Treatment outcomes in the unit TB registers were not documented for 13.9% of TB patients and follow-up sputum smear examination results were not available for more than 30% of smear-positive pulmonary TB patients. This evidence indicates that health workers were not properly recording the required patient data in the unit TB registers. Lack of health workers' capacity and poor perceptions about the importance of assuring data quality were possible reasons suggested by respondents. Inappropriate documentation of data seriously affects TB data quality, that is, accuracy, reliability, completeness, timeliness, and integrity of the data [29]. We suggest that strengthening or establishing internal mentoring is necessary. For example, internal mentoring using District Health Offices monitoring and evaluation officers and TB control program coordinators may be a practical and less resource intensive approach to improve data quality. In addition, adequate time allocation and attention for monitoring and evaluation part of the standard TB training may also be helpful.

### 4.4. Health Workers Lack of Adherence to TB Treatment Guideline

Supervised treatment, which includes direct observation of therapy, helps patients to regularly take their drugs and complete treatment, thus achieving cure and prevention of drug resistant TB [30]. As majority of the respondents said, DOTS was not properly implemented in health facilities. Health workers were not managing TB patients by adhering to the treatment guideline according to DOTS. Similarly, a former study showed health worker's poor adherence to the TB treatment guideline [11, 31]. This may be related to lack of understanding among health workers about the consequences of not adhering to DOTS during TB treatment. As the TB control program coordinators indicated, this may have resulted due to health workers' negligence to adhere to the treatment guideline. Provision of regular-refresher training on TB, strengthening of regular program monitoring and evaluation, and supportive supervision may be useful to appropriately implement DOTS in the study area.

### 4.5. Diagnostic Tool Shortage

In high-incidence countries, TB control relies on passive case finding among individuals self-presenting to health care facilities, followed by either diagnosis based on clinical symptoms or laboratory diagnosis using sputum smear microscopy which has very low sensitivity [32]. Inability to rapidly diagnose and treat TB patients leads to increased morbidity, mortality, and ongoing transmission of TB [33]. Like other developing countries, most peripheral health facilities in Ethiopia widely use sputum smear microscopy to diagnose TB [17]. This study highlighted lack of diagnostic tool at the peripheral health facilities of the study zone for laboratory diagnosis of PTB- and EPTB cases. The TB program coordinators acknowledged this as a reason for diagnostic delay and low TB case detection in their context. Globally, emphasis has been given for timely diagnosis and treatment of all TB cases [34]. However, accurate TB diagnosis is difficult, particularly in peripheral health facilities where there are limited diagnostic tools and poor clinical experiences among health workers. In such situations, PTB- and EPTB suspects are often referred to hospitals and higher level private health institutions where better diagnostic tools are available. We suggest that improving the diagnostic capacity by availing better diagnostic tools for TB at peripheral health institutions expedites timely diagnosis and treatment of TB.

Our study has limitations. We only assessed the challenges of TB control program performance in the study area from TB control program coordinators' and health workers' perspectives. Because of limited time and resource, we did not assess the challenges from the patients' perspectives. Otherwise, this descriptive qualitative study provides valuable insights regarding the health system challenges of the TB control program in the study zone from a range of different health care providers' and TB control program coordinators' perspectives. We believe that the suggested recommendations may contribute to the improvement of TB control program performance in the study area.

## 5. Conclusions

From health workers' and TB control program coordinators' perspectives, the study identified a number of challenges affecting the success of TB control program performance in the study area. Most of the challenges were health system based and require adequate attention and intervention by health authorities in the study area. Among others, ensuring uninterrupted anti-TB drugs and laboratory reagents supplies to all health facilities in the study area is essential for the success of the TB control program performance. In addition, the health authorities should develop and implement a sound retention strategy to attract and motivate health professionals to work in rural and remote health facilities. Regular training of health workers on standard TB care and health management information system is mandatory. Moreover, improving the diagnostic capacity by availing better diagnostic tools at peripheral health facilities expedites timely diagnosis and treatment of TB. Last but not least, regular monitoring and evaluation of program performance is imperative to identify the real challenges and improve TB control program performance in the study area.

## References

[1] World Health Organization (2015). *Global Tuberculosis Report 2015*.

[2] Gebreegziabher S. B., Yimer S. A., Bjune G. A. Tuberculosis case notification and treatment outcomes in West Gojjam Zone, Northwest Ethiopia: a five-year retrospective study. *Journal of Tuberculosis Research*.

[3] World Health Organization (WHO) (2008). Stop TB policy paper: contributing to health system strengthening; guiding principles for national tuberculosis programmes.

[4] Tadesse T., Demissie M., Berhane Y., Kebede Y., Abebe M. (2013). Long distance travelling and financial burdens discourage tuberculosis DOTs treatment initiation and compliance in Ethiopia: a qualitative study. *BMC Public Health*.

[5] Gele A. A., Sagbakken M., Abebe F., Bjune G. A. (2010). Barriers to tuberculosis care: a qualitative study among Somali pastoralists in Ethiopia. *BMC Research Notes*.

[6] Sagbakken M., Frich J. C., Bjune G. (2008). barriers and enablers in the management of tuberculosis treatment in addis ababa, ethiopia: a qualitative study. *BMC Public Health*.

[7] Xu W., Lu W., Zhou Y., Zhu L., Shen H., Wang J. (2009). Adherence to anti-tuberculosis treatment among pulmonary tuberculosis patients: a qualitative and quantitative study. *BMC Health Services Research*.

[8] Soomro M. H., Qadeer E., Mørkve O. (2013). Barriers in the management of tuberculosis in Rawalpindi, Pakistan: a qualitative study. *Tanaffos*.

[9] Ayisi J. G., Van’t Hoog A. H., Agaya J. A. (2011). Care seeking and attitudes towards treatment compliance by newly enrolled tuberculosis patients in the district treatment programme in rural western Kenya: a qualitative study. *BMC Public Health*.

[10] Hasker E., Khodjikhanov M., Sayfiddinova S. (2010). Why do tuberculosis patients default in Tashkent City, Uzbekistan? A qualitative study. *International Journal of Tuberculosis and Lung Disease*.

[11] Amo-Adjei J. (2013). Views of health service providers on obstacles to tuberculosis control in Ghana. *Infectious Diseases of Poverty*.

[12] Cowan J. F., Cowan J. G., Barnhart S. (2013). A qualitative assessment of challenges to tuberculosis management and prevention in Northern Ethiopia. *International Journal of Tuberculosis and Lung Disease*.

[13] Cattamanchi A., Miller C. R., Tapley A. (2015). Health worker perspectives on barriers to delivery of routine tuberculosis diagnostic evaluation services in Uganda: a qualitative study to guide clinic-based interventions. *BMC Health Services Research*.

[14] Central Statistics Autority of Ethiopia (2008). *Summary and Statestical Report of the 2007 Population and Housing Census*.

[15] Braun V., Clarke V. (2006). Using thematic analysis in psychology. *Qualitative Research in Psychology*.

[16] Shiferaw M. B., Hailu H. A., Fola A. A. (2015). Tuberculosis laboratory diagnosis quality assurance among public health facilities in west Amhara region, Ethiopia. *PLoS ONE*.

[17] Getachew T., Bekele A., Defar A. (2015). Tuberculosis service provision in Ethiopia: health facility assessment. *American Scientific Research Journal for Engineering, Technology, and Sciences*.

[18] Inotu A., Abebe F. (2014). Assessment of defaulting from directly observed treatment short course (DOTS) and its determinants in Benin City, Nigeria. *Journal of Tuberculosis Research*.

[19] Nezenega Z. S., Gacho Y. H. M., Tafere T. E. (2013). Patient satisfaction on tuberculosis treatment service and adherence to treatment in public health facilities of Sidama zone, South Ethiopia. *BMC Health Services Research*.

[20] Federal Democratic Republic of Ethiopia Ministry of Health (2012). *Guidelines for Clinical and Programmatic Management of TB, Leprosy and TB/HIV in Ethiopia*.

[21] Gomes V. F., Andersen A., Lemvik G. (2013). Impact of isoniazid preventive therapy on mortality among children less than 5 years old following exposure to tuberculosis at home in Guinea-Bissau: a prospective cohort study. *BMJ Open*.

[22] Ayieko J., Abuogi L., Simchowitz B., Bukusi E. A., Smith A. H., Reingold A. (2014). efficacy of isoniazid prophylactic therapy in prevention of tuberculosis in children: a meta-analysis. *BMC Infectious Diseases*.

[23] Shewarega A., Paul D., Welelaw N., Sami T., Yared Y. (2015). *Ethiopia: National Survey of the Integrated Pharmaceutical Logistics System*.

[24] The Federal Democratic Republic of Ethiopia Pharmaceuticals Fund and Supply Agency (PFSA) (2014). *Integrated Pharmaceutical Logistics System: Changing the Supply Chain System of Ethiopia to Impact the Health Outcomes*.

[25] Mesfin M. M., Newell J. N., Walley J. D. (2009). Quality of tuberculosis care and its association with patient adherence to treatment in eight Ethiopian districts. *Health Policy and Planning*.

[26] Ibrahim L. M., Hadjia I. S., Nguku P. (2014). Health care workers’ knowledge and attitude towards TB patients under Direct Observation of Treatment in Plateau state Nigeria, 2011. *Pan African Medical Journal*.

[27] Wynne A., Richter S., Banura L., Kipp W. (2014). Challenges in tuberculosis care in Western Uganda: health care worker and patient perspectives. *International Journal of Africa Nursing Sciences*.

[28] World Health Organization (WHO) (2010). *Increasing Access to Health Workers in Remote and Rural Areas Through Improved Retention. Global Policy Recommendations*.

[29] Ledikwe J. H., Grignon J., Lebelonyane R. (2014). Improving the quality of health information: a qualitative assessment of data management and reporting systems in Botswana. *Health Research Policy and Systems*.

[30] World Health Organization (2010). *The Global Plan to Stop TB 2011–2015: Transforming the Fight towards Elimination of Tuberculosis*.

[31] Mala G., Moser A., Dinant G.-J., Spigt M. (2014). Why tuberculosis service providers do not follow treatment guideline in Ethiopia: a qualitative study. *Journal of Evaluation in Clinical Practice*.

[32] Parsons L. M., Somoskövi Á., Gutierrez C. (2011). Laboratory diagnosis of tuberculosis in resource-poor countries: challenges and opportunities. *Clinical Microbiology Reviews*.

[33] Yusuf N. W., Iram S., Zeenat A., Hussain S., Aslam M. (2015). Rapid diagnosis of tuberculosis using Xpert MTB/RIF assay—report from a developing country. *Pakistan Journal of Medical Sciences*.

[34] World Health Organization (WHO) (2007). Improving the diagnosis and treatment of smear-negative pulmonary and extra pulmonary tuberculosis among adults and adolescents. Recommendations for HIV-prevalent and resource-constrained settings.

